# Tree-based machine learning methods for predicting vehicle insurance claim size

**DOI:** 10.3389/fdata.2026.1778363

**Published:** 2026-03-23

**Authors:** Edossa Merga Terefe, Merga Abdissa Aga

**Affiliations:** 1 Department of Diagnostics and Intervention, Umeå University, Umeå, Sweden; 2 Research Center for Statistics, University of Geneva, Geneva, Switzerland; 3 Department of Statistics, Salale University, Fiche, Ethiopia

**Keywords:** claim size prediction, machine learning, tree-based ensemble methods, variable importance, vehicle insurance

## Abstract

Vehicle insurance claim severity modeling requires accurate and interpretable methods that can handle skewed and heterogeneous loss data. This study provides a structured empirical comparison between classical parametric regression models and tree-based ensemble learning approaches for predicting claim size conditional on claim occurrence. The analysis is conducted within a cross-sectional conditional severity framework using real-world motor insurance data. We implement and compare ordinary least squares (OLS), a Tweedie generalized linear model (GLM), and three ensemble methods: bagging, random forests (RFs), and gradient boosting. Model performance is evaluated using out-of-sample root mean square error (RMSE), and variable importance measures assess the relative contribution of predictors. The results indicate that tree-based ensemble methods achieve modest improvements in predictive accuracy relative to classical parametric models. The Tweedie GLM remains a competitive, flexible parametric benchmark for skewed positive claim amounts. Variable importance analysis consistently identifies premium and insured value as key determinants of claim severity. Overall, the findings suggest that ensemble learning methods can complement traditional actuarial models, offering additional flexibility in capturing non-linear effects while maintaining comparable predictive performance in moderate-complexity severity data.

## 1 Introduction

A central challenge in the insurance industry is to determine premiums that accurately reflect the level of risk represented by each policyholder. Risk heterogeneity across customers necessitates a comprehensive understanding of factors that influence both the likelihood and the financial magnitude of insurance claims. In non-life insurance, particularly motor vehicle insurance, accurate estimation of claim severity is essential for pricing, capital allocation, and risk management. Underestimation of claim costs may threaten insurer solvency, whereas overestimation can reduce competitiveness and impair customer retention (Hanafy and Ming, 2021).

While premium determination typically involves modeling both claim frequency and claim severity, the present study focuses exclusively on conditional claim severity, defined as the claim size given that a claim has occurred. This distinction is important: rather than modeling the full frequency–severity process or the unconditional loss distribution, we restrict attention to strictly positive claim amounts and evaluate predictive performance within a conditional framework.

Recent developments in insurance analytics have demonstrated the growing relevance of advanced predictive modeling techniques. Studies similar to those by (Guillen et al. 2019), (Roel et al. 2017), (Gao and Wuthrich 2018), and (Gao et al. 2019) illustrate the increasing integration of telematics and machine learning (ML) tools into actuarial practice. These contributions emphasize the role of flexible modeling approaches in capturing complex relationships within high-dimensional insurance data.

Parallel to this development, actuarial research has explored tree-based methods in the context of individual-claims reserving and structured loss development. For example, Janoušek and PeŽta (2025) investigates regression trees and bagging techniques for individual-claims reserving, highlighting issues of predictive stability and robustness. Similarly, (Maciak et al. 2022) proposes functional profile methods for structured claims development modeling, emphasizing interpretability and the longitudinal structure of insurance data.

However, reserving frameworks differ fundamentally from cross-sectional severity modeling. Reserving models typically rely on dynamic claim development processes, structured development triangles, reporting delays, and time-evolving liabilities. In contrast, the present study employs a cross-sectional dataset of settled claims and does not model claim development, outstanding liabilities, or temporal updating. Although methodological insights regarding stability and interpretability are relevant across contexts, structured development modeling does not directly carry over to the conditional severity setting considered here.

The dataset analyzed in this study comprises motor insurance contracts from Ethiopia and exhibits substantial zero inflation, with the majority of policies reporting no claim payment during the observation period. To maintain coherence with the modeling objective, we conduct a conditional severity analysis by excluding observations with zero claims and focusing exclusively on strictly positive claim sizes. This approach allows for a targeted comparison of predictive models for claim severity without conflating frequency and severity components.

Within this framework, we provide a structured empirical comparison between classical parametric regression models and tree-based ensemble learning methods. Specifically, we employ a Tweedie generalized linear model (GLM) as a flexible parametric benchmark for skewed positive claim amounts and compare its performance with bagging, random forests (RFs), and gradient boosting. Model evaluation is based on out-of-sample predictive accuracy and interpretability considerations.

Rather than proposing a novel methodological framework, this study contributes empirical evidence on the comparative performance of established statistical and machine learning approaches when applied to motor insurance severity data from an emerging market context. Given the relative scarcity of publicly analyzed Ethiopian insurance datasets in the actuarial literature, the findings provide practical insights into the behavior, stability, and predictive capacity of different modeling strategies under data-constrained conditions.

The remainder of the article is organized as follows. Section 2 describes the dataset and presents exploratory analysis. Section 3 outlines the statistical and machine learning models. Section 4 reports empirical results and model comparisons. Section 5 concludes with a discussion and implications for practice.

## 2 Dataset and exploratory analysis

### 2.1 The data

The data used for this analysis were obtained from a large database of the Ethiopian Insurance Corporation, one of the biggest insurance companies in Ethiopia. The Ethiopian vehicle insurance dataset section provided a portion of the data. It consists of policy and claim information for individual-level vehicle insurance. The dataset originally contains *n* = 802, 036 individual contracts, represented by the observations (*X*_1_, *Y*_1_), …, (*X*_*n*_, *Y*_*n*_) where $X=\left(X_{1},\ldots ,X_{p}\right)\in {\mathbb{R}}^{p}$ denotes a vector of *p* = 10 predictors, and *Y* ∈ ℝ denotes the response variable representing the claim size. The data were corresponded to the period between July 2011and June 2018. The different predictors used in the analysis are summarized in Table 1.

**Table 1:** Description of predictors in Ethiopian vehicle insurance dataset.

**S. No**.	**Name**	**Type**	**Domain/Levels**	**Description/representation**
1	Sex	Categorical	0, 1, 2	0 = legal entity, 1 = male, 2 = female
2	Insurance type	Categorical	1201, 1202, 1204	1201 = private, 1202 = commercial, 1204 = motor trade road risk
3	Vehicle type	Categorical	pick-up, truck, bus, ...	Type of vehicle grouped into six categories
4	Usage	Categorical	fare paying passengers, taxi, general cartage, ...	A usual usage of the vehicle grouped into six categories
5	Make	Categorical	Toyota, Isuzu, Nissan,...	Manufacturer company
6	Coverage	Categorical	comprehensive, liability	Scope of the insurance.
7	Production year	Integer	1960–2018	Vehicle's production year
8	Insured value	Continuous	ℝ+	Vehicle's price in USD
9	Premium	Continuous	ℝ+	Premium amount in USD

The terms, liability and comprehensive coverage in Table 1 are defined as:

- Comprehensive coverage: The company covers all the losses that happen to the car whenever the conditions of the agreement are satisfied.
- Liability or third-party coverage: The car can cause damage to someone or someone's property. If the policyholder already has liability coverage in place, the insurance company covers the costs in this case. Liability amount, which a policyholder has to pay as a part of the premium, is nationally fixed almost every year for each car type across all the insurance companies operating in the country. Liability cases are usually taken to court or settled through negotiation. However, the affected party should not be either a family member or a relative of the policyholder.

Computation of the premium, which is transformed to a logarithmic scale in the analysis, is determined as a function of:

- Insured value, transformed to a logarithmic scale.
- Production year: For the first 3 years, the age of the car is not considered, but after 3 years, the age loader computation technique, which takes into account the age of the vehicle, is applied.
- No claim discount (NCD): Upon the renewal of the contract after a year, a policyholder gets a 20% discount from the previous year's premium and adjusted for inflation if the policyholder has not applied for a claim in that year. The policyholder can get up to 60% of discount in consecutive years, but it is reduced by the vehicle's age.
- Contingency, Plant and Machinery (CPM): Applicable for those cars that are operated under different circumstances. For instance, the premium for loader-type vehicles can be computed assuming they are at engineering (construction) sites; however, if the vehicle is involved in a road accident, the computation requires additional consideration, and the mechanism differs.

Some predictors, such as carrying capacity and seat number, are removed from the dataset prior to analysis and modeling because they are not coded correctly.

### 2.2 Claim size variable

In our analysis, the claim size is a continuous response variable *Y* ∈ ℝ. It is originally the amount in Ethiopian currency, the Birr, and it is converted to USD during data analysis. The distribution of the response variable *Y* is strongly zero-inflated, since about 92.5% of contracts have no claim size. Thus, instead of visualizing and modeling this distribution directly, we first select the non-zero observations. Given that the claim has to be paid for policyholder *i*, it is determined as


$$\begin{array}{l} Y_{i}=\text{claimsize}=\frac{{\text{Insuredvalue}}_{i}}{{\text{Marketvalue}}_{i}}\times {\text{Loss}}_{i}, \end{array}$$ (1)

where

- Market value is the market price of the car when it was bought. The insurer collects the market price for each car type almost every year. The data on the market value are taken either from importers or informally from other institutions. It can be either more or less than the insured value. Knowing the car's market value helps adjust the claim amount so it is not too high if the insured value is unreliable. In most cases, insured value and market value are the same.
- Loss: When an accident happens, the damaged vehicle is inspected by the engineering experts who work in the insurance company. These experts, known as server decision makers, examine each part of the vehicle, identify the affected parts, and propose either replacing or fixing the vehicle. Once the affected parts have been identified, the server determines their price, and a bid to repair the damaged car is published. Including the server decision members, anyone who has a license to do so can usually participate in the bid competition. The loss is determined as follows.

In some cases, the amount of the claim may exceed either the insured value or the market price. It is mandatory to have at least liability insurance coverage for all vehicles under a country's regulations, even if comprehensive insurance is for the vehicle's owner's safety. Additionally, a policyholder can have Bandit, Shifta, and Gorilla (BSG) and Passengers' legal liability (PLL) insurance coverage, but only if comprehensive coverage is secured first. The terms BSG and PLL are defined as:

- Bandit, Shifta, and Gorilla (BSG): A contract agreement in case the car is robbed or stolen. To the best of the insurer's knowledge, it is a vehicle insurance component applicable only in Ethiopia.
- Passengers' legal liability (PLL): This is applicable for fare-paying passengers, in case someone is affected by an accident while in the car. Similar to liability coverage, its amount is fixed and paid as part of the premium, and the insurer would pay up to 40,000 birr to a passenger in case of an accident.

Even though both BSG and PLL insurance coverage depend on the interest of the policyholder and they are optional, applicable if and only if the comprehensive insurance agreement is to take place or has already taken place.

The insured value in Equation 1 does not include the values of liability, BSG, and PLL, even though they are included in the contract. It contains only the value of comprehensive coverage. Thus, claim size can be larger than the insured value if:

- (total) loss + (liability insurance) + (PLL) > insured value,

where total loss is an overall loss of the car due to a severe accident, and it is impossible to repair. In that case, the insurance company pays exactly the insured value as a claim.

### 2.3 Data pre-processing

The pre-processing steps are summarized in Table 2 and were applied prior to model estimation.

**Table 2:** Data pre-processing steps.

**Step**	**Description**
Zero claims	Observations with zero claim amounts were removed to focus on claim severity conditional on claim occurrence
Missing values	Data were checked for missing values; where present, appropriate imputation methods were applied
Categorical variables	Converted into dummy variables using one-hot encoding prior to model fitting
Train–test split	Dataset randomly divided into 70% training set and 30% testing set for out-of-sample evaluation
Scaling	Continuous variables were retained on original scale; scaling applied only where required for model stability

### 2.4 Exploratory data analysis

To make assumptions about the data and find a model that fits it best, it is important to carry out an exploratory data analysis (EDA), since it has a significant role in letting the data speak for themselves prior to or as part of a formal analysis. It allows the researcher to influence and criticize an intended analysis. Additionally, EDA techniques may reveal additional information that may not be directly related to the research question. For example, EDA could suggest fruitful new lines of research (Maindonald and Braun, 2010).

The purpose of statistical graphics is to provide visual representations of quantitative and qualitative information. As a methodological tool, statistical graphics comprises a set of strategies and techniques that provide researchers with important insights about the data under examination and help guide the subsequent steps of the research process. The objectives of graphical methods are to explore and summarize the contents of large and complicated datasets, address questions about the variables in an analysis (for example, the distributional shapes, ranges, typical values, and unusual observations), reveal structure and pattern in the data, check assumptions in statistical models, and facilitate greater interaction between the researcher and the data. Various graphical methods were examined to visualize data in raw and amalgamated formats.

The most widely recognized graphical tool to display and examine the frequency distribution and the density of a single continuous variable is the histogram.

**Figure 1:**
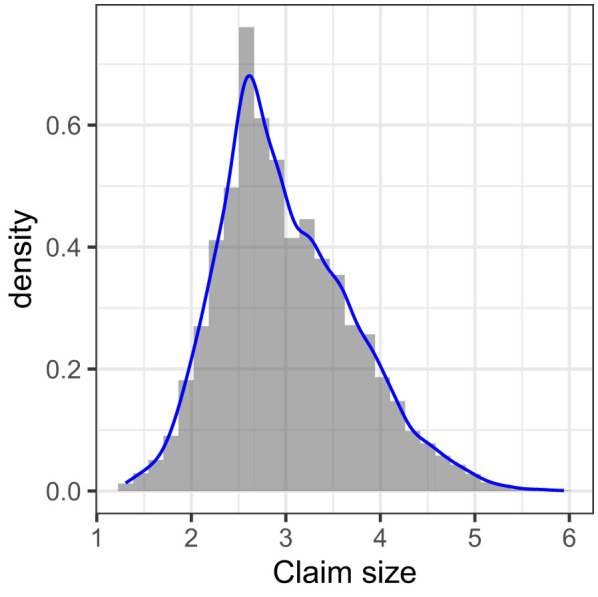
Frequency histogram and superimposed density plot representations of the natural logarithm of the claim size distribution. The distribution of the response variable *Y* is strongly zero-inflated, since only about 7.5% of the contracts have a non-zero claim payment. Thus, in our analysis, instead of visualizing and modeling this distribution directly, we consider only policyholders who have ever received a positive claim.

Another common tool to visualize the observed distribution of data is to plot a smoothed histogram, commonly referred to as an empirical density, with the blue curve superimposed on the histogram in Figure 1. In constructing the histogram, the bin width was selected using the Freedman–Diaconis rule, which adapts the number of bins to both the sample size and the variability of the data, thereby reducing the arbitrariness in bin selection (Freedman and Diaconis, 1981). The histogram was scaled so that the total area equals 1, allowing direct comparison with probability density functions. However, histograms can be sensitive to the choice of bin width and bin boundaries, which may substantially affect their shape. To address this limitation, we also present the empirical density, estimated using a kernel smoothing method (Silverman, 1986). Unlike histograms, kernel density estimation provides a continuous approximation of the underlying distribution, offering a smoother, more stable visualization of the data's distributional characteristics.

### 2.5 Exploring relationships between covariates and claim size

Relationships between the predictors and the response variable can be depicted by graphical methods. Side-by-side boxplots are one way of graphically displaying the relationship between qualitative and quantitative variables. It is an excellent tool for conveying location and variation information in datasets, particularly for detecting and illustrating changes in location and variation across different groups of data.

**Figure 2:**
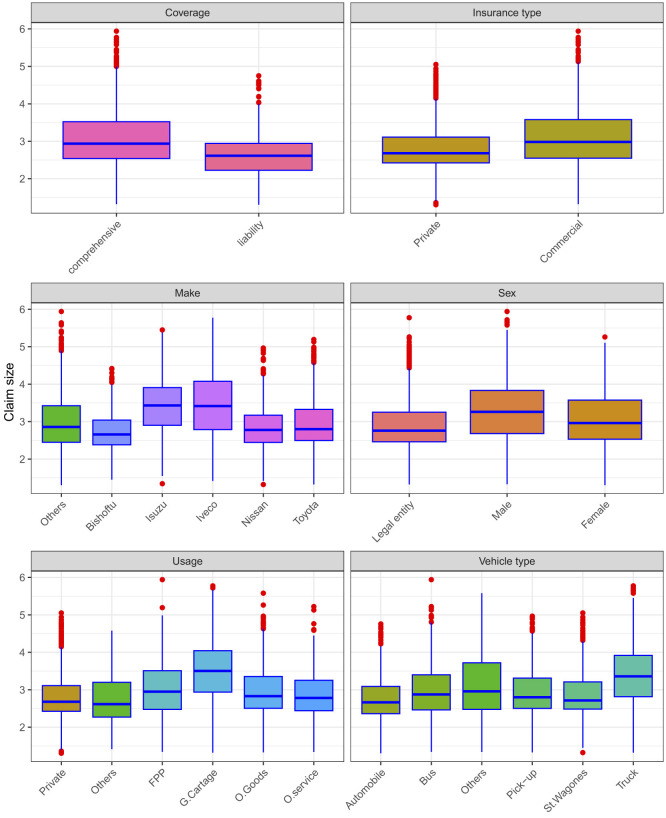
Boxplots of the natural logarithm of claim size against qualitative predictors.

The boxplots of claim size against the different qualitative predictors are shown in Figure 2. Several predictors exhibit noticeable heterogeneity across their categories. For instance, in the boxplots of the log of claim size against sex, clear differences appear among the three groups of sex. Male policyholders tend to have higher claim payments than either their female counterparts or legal entities. Similarly, differences in claim size are observed in vehicle usage, with vehicles used for general cartage showing the highest claim payments, followed by vehicles carrying fare-paying passengers. Moreover, vehicles manufactured by Isuzu and Iveco tend to incur higher claim payments than those produced by other companies, consistent with the insurer's prior identification of these brands as relatively risky. Differences across groups are also evident in other covariates such as vehicle type, insurance type, and insurance coverage.

Boxplots are not only effective for visualizing group-wise differences but also for assessing the spread and detecting potential outliers in the data. Given the large sample size in this dataset, the appearance of a considerable number of outliers is statistically expected since larger samples naturally increase the probability of observing extreme values. In this context, the relative frequency of outside values in the boxplots does not necessarily imply data quality issues or true anomalies but reflects the inherent variability in large insurance claim datasets. This is consistent with the statistical property that, even under a normal distribution, approximately 0.7% of values are expected to fall outside the whiskers of a boxplot (beyond 1.5 × interquartile range[IQR]).

In our data, the relative proportion of outside values ranged from 0 to 2.8%. Comparing these proportions with what would be expected from a comparable normal or Tweedie-like distribution, the observed deviations are within plausible probabilistic bounds. This indicates that the data are consistent with the heavy-tailed nature commonly observed in claim amounts, in which extreme but valid observations occur more frequently than under a purely normal model. Hence, while the presence of numerous external values highlights heterogeneity and heavy-tailed behavior in claim payments, it also reaffirms the appropriateness of using flexible models, such as the Tweedie GLM, for such data.

Additionally, examining group-specific variability shows that claim payments for male policyholders are more dispersed than those for females or legal entities. Vehicles used for general cartage and fare-paying passengers also show wider spreads than those in other categories. Likewise, heterogeneity in variance is observed across groups defined by insurance coverage, insurance type, manufacturer company, and vehicle type, reflecting structural differences in claim risk profiles across these factors.

Analogous to boxplots, Scatterplots are an obvious way to visualize a relationship between two quantitative variables that are numerically comparable. They are useful as a preliminary step before performing a regression analysis.

**Figure 3:**
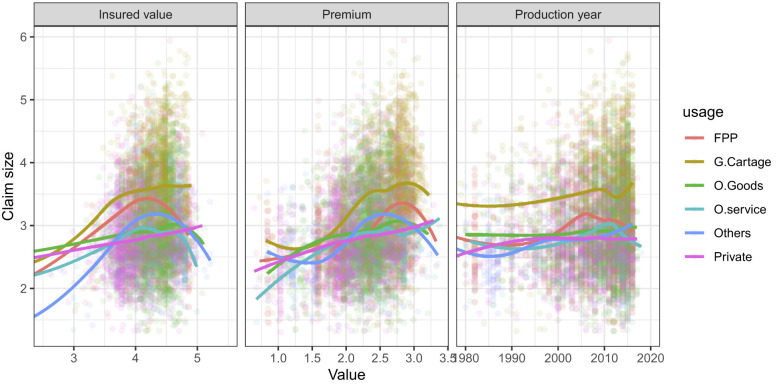
Scatterplots matrix: a bivariate profiling of relationships between claim size and quantitative predictors.

Figure 3 shows that scatterplots are a bivariate relationship between claim size and quantitative predictors. It is difficult to detect clear trends in any of the plots. However, when stratifying by usage predictor groups, we observe differences in claim size across groups.

In addition to the scatterplot matrix seen in Figure 3, we computed correlation coefficients between claim size and insured value, premium, and production year as 0.22, 0.33, and 0.11, respectively. Even though none of the coefficients between claim size and the covariates are considered to be strong, there are some notable associations. For instance, claim size appears to have a moderate positive correlation with insured value, premium, and production year, meaning that as vehicles' insured value, premium, and production year increase, their claim payments tend to increase as well. Correlation coefficient-based relationships are usually teased out more clearly when building the (final) model.

The *loess curves* drawn on top of the scatterplots indicate a possibly non-linear relationship between the two variables. The curves for claim size against insured value and premium are upside-down U-shaped, peaking around the middle of the insured value and premium ranges for most groups of usage predictors. This means that vehicles with moderate insured value and/or moderate premium have larger claim sizes than those with lower and higher insured value and/or premium. Because this trend is non-linear, this finding could not have been inferred from the correlations alone. In contrast, when we consider the private group of usage predictors, the relationship between claim size and insured value appears linear, with a positive slope.

## 3 Statistical methods

### 3.1 Tweedie family

The Tweedie family of distributions belongs to the exponential dispersion family (EDF) and provides a flexible framework for modeling non-negative and right-skewed data (Jørgensen, 1997; Dunn and Smyth, 2005). The probability density function of a Tweedie-distributed random variable *Y* is given by:


$$f\left(y;\mu ,\phi ,p\right)=a\left(y,\phi ,p\right)\exp\left[\frac{1}{\phi }\left(y\frac{\mu^{1-p}}{1-p}-\frac{\mu^{2-p}}{2-p}\right)\right],$$

for *y*>0, where μ is the mean, ϕ>0 is the dispersion parameter, *p* is the power parameter, and *a*(*y*, ϕ, *p*) is a normalizing constant ensuring the density integrates to one.

The variance–mean relationship of the Tweedie family is defined as:


$$\text{Var}\left(Y\right)=\phi \mu^{p}.$$

Different values of *p* yield well-known special cases:


$$\{\begin{array}{ll} p=0 & \text{Normal distribution},\\ p=1 & \text{Poisson distribution},\\ p=2 & \text{Gamma distribution},\\ p=3 & \text{Inverse Gaussian distribution}. \end{array}$$

(Jørgensen, 1987; Smyth and Jørgensen, 2002).

For *p* ∈ (1, 2), the Tweedie distribution corresponds to a compound Poisson-Gamma model frequently used in frequency-severity modeling. However, in the present study, the analysis is restricted to strictly positive claim sizes. Therefore, the compound Poisson interpretation is not invoked. Instead, we use the Tweedie family as a flexible parametric class for continuous, positive, and heteroscedastic data (Dunn and Smyth, 2008).

When *p*>2, the distribution behaves similarly to Gamma and inverse Gaussian models while allowing a more flexible variance–mean relationship. This flexibility makes it suitable as a benchmark model for heavy-tailed insurance claim severity data (Dunn and Smyth, 2008).

#### 3.1.1 Conditional severity modeling framework

The original portfolio exhibits substantial zero inflation (approximately 92.5% zero claims). To ensure coherence between the distributional assumption and the modeling objective, the analysis is conducted within a conditional severity framework:


$$\begin{array}{l} Y_{i}|\left(Y_{i}>0\right) \end{array}$$

Thus, the response variable consists exclusively of strictly positive claim size, and the model estimates the conditional mean severity rather than the unconditional loss distribution. This distinction is important, as the model does not attempt to represent claim frequency or compound loss processes.

#### 3.1.2 Model specification

A Tweedie generalized linear model (GLM) with log-link function was fitted:


$$\text{log}\left(\mu_{i}\right)=X_{i}\beta ,$$

where:


$$\mu_{i}=Y_{i}|\left(Y_{i}>0\right).$$

*X*_*i*_ is the vector of explanatory variables and β is the vector of regression coefficients. The log-link ensures that predicted claim severities are positive and allows multiplicative interpretation of covariate effects.

The power parameter *p* was selected empirically via grid search over plausible values *p* ∈ [2, 3], guided by residual diagnostics and out-of-sample predictive performance. The selected value (approximately *p* = 2.5) reflects the heavy-tailed, heteroscedastic nature of the positive claim severity data.

Model adequacy was evaluated using residual analysis and information criteria. Predictive performance was assessed using the root mean square error (RMSE) on the held-out test dataset.

### 3.2 Review of machine learning methods

Machine learning (ML) is now well established in many areas. In contrast to the statistical modeling approach, ML algorithms do not assume any specific model structure for the data. ML methods capture the underlying structure of data and are therefore more efficient in handling large data with arbitrary degrees of complexity. One major task of ML is to construct good models from datasets.

Among ML algorithms, ensemble methods are a common choice for analyzing large and complex datasets. Originally developed to reduce variance—thereby improving the accuracy of automated decision-making systems—ensemble methods have since been successfully applied to a variety of problems (Zhang and Ma, 2012), such as predictor selection, class-imbalanced data, confidence estimation, and error correction.

The main idea of the ensemble methodology is to weigh several individual learners and combine them to obtain a learner that outperforms most of them. In fact, combining learner outputs does not necessarily lead to performance that is guaranteed to be better than the best learner in the ensemble. Rather, it reduces the likelihood of relying on a poorly performing learner. Ensemble methods mimic the idea of seeking several opinions before making a crucial decision: individual opinions are weighted and combined to reach the final decision (Polikar, 2006).

A general principle of ensemble methods is to construct a linear combination of some model-fitting method, instead of using a single fit, to improve predictive performance. More precisely, consider an estimation of a real-valued function


$$f:{\mathbb{R}}^{p}\to \mathbb{R}$$

based on data (*x*_*i*_, *y*_*i*_), *i* = 1, …, *n*, where *x* is a *p*-dimensional predictor variable and *y* is a univariate response. We may then generalize to functions *f*(*x*) and other data types.

Given some input data, we learn several functions ${\hat{f}}_{1},{\hat{f}}_{2},\ldots ,{\hat{f}}_{B}$, called learners, by reweighting the input data. We can then construct an ensemble-based estimate ${\hat{f}}_{\text{ens}}\left(x\right)$ by taking linear combinations of the individual learners as an additive expansion (Elish, 2009):


$$\begin{array}{l} {\hat{f}}_{\text{ens}}\left(x\right)=\overset{B}{\underset{i=1}{\sum }}w_{i}{\hat{f}}_{i}\left(x\right), \end{array}$$ (2)

where the ${\hat{f}}_{i}\left(x\right)$ are estimates obtained from the *i*th reweighted dataset and *w*_*i*_ are the linear combination coefficients. For instance, *w*_*i*_ = 1/*B* in bagging (see Section 3.2.1) and boosting (see Section 3.2.3).

In this study, three ensemble learning algorithms—bagging, random forest, and boosting—are considered. For model performance comparison, two non-ensemble learning techniques, namely, ordinary linear regression and decision trees, are also applied to an Ethiopian vehicle insurance dataset to predict claim size.

#### 3.2.1 Bagging

Bagging (Breiman, 1996), short for bootstrap aggregating, is an ensemble method for improving unstable estimation or classification schemes. The two key ingredients of Bagging are bootstrap and aggregation.

Bagging uses the bootstrap distribution for generating different learners. Specifically, it applies bootstrap sampling (Efron and Tibshirani, 1993) to obtain subsets of the data for training. Given an original dataset, a new dataset containing *n* training observations is generated by sampling with replacement. Some observations may appear multiple times, while others may be absent. By repeating this *B* times, *B* bootstrap samples are obtained. A learner is then trained on each sample.

Bagging adopts standard strategies for aggregating learners' outputs: voting for classification and averaging for regression. To predict a test instance in regression, Bagging feeds the instance to its learners, collects their outputs, and averages them. Formally, a learner ${\hat{f}}_{i}\left(x\right)$ is fitted on each of the *B* bootstrapped samples, and the average prediction is computed as:


$$\begin{array}{l} {\hat{f}}_{\text{avg}}\left(x\right)=\frac{1}{B}\overset{B}{\underset{i=1}{\sum }}{\hat{f}}_{i}\left(x\right), \end{array}$$

resulting in a single low-variance learner.

Mathematically, bagging can be viewed as reducing the variance component of the prediction error decomposition:


$$\text{MSE}\left(x\right)=\mathrm{Bias}{\left[\hat{f}\left(x\right)\right]}^{2}+\mathrm{Var}\left[\hat{f}\left(x\right)\right]+\sigma^{2}.$$

By averaging over multiple bootstrapped models, the expected variance term $\mathrm{Var}\left[\hat{f}\left(x\right)\right]$ decreases approximately in proportion to 1/*B*, leading to improved generalization.

#### 3.2.2 Random forest

Random Forest (RF) is a state-of-the-art ensemble method developed by Breiman and Cutler (2008), and it has become a widely used technique in data science (Dangeti, 2017). It is an extension of Bagging, with the key innovation being randomized predictor selection. During the construction of a decision tree, RF randomly selects a subset of predictors at each split, and the best split is then chosen from this subset.

RF reduces the variance of individual trees by aggregating many trees. Approximately one-third of the training samples are excluded from each bootstrap sample; these are called “out-of-bag” (OOB) samples. Predictions on OOB samples provide an internal error estimate. For regression, the final prediction is the average of predictions across trees, while for classification it is the majority vote or average predicted probability (Jones and Linder, 2016).

Formally, if ${\hat{f}}_{b}\left(x\right)$ is the prediction from the *b*^*th*^ tree trained with random feature subset Θ_*b*_, the RF predictor is given by:


$${\hat{f}}_{\text{RF}}\left(x\right)=\frac{1}{B}\overset{B}{\underset{b=1}{\sum }}{\hat{f}}_{b}\left(x;{\text{Θ}}_{b}\right).$$

The randomization of Θ_*b*_ ensures the decorrelation of trees, thereby reducing the ensemble variance while maintaining low bias. Theoretical analysis shows that the generalization error converges as the number of trees *B* → ∞, provided each tree has a positive margin over random guessing.

#### 3.2.3 Gradient boosting

Boosting algorithms were introduced in ML by Schapire (1990) and Freund (1995). The term “boosting” refers to a family of algorithms that convert weak learners (slightly better than random guessing) into strong learners (close to perfect performance). The method sequentially fits a series of models, each correcting errors of its predecessor, and combines them into an ensemble with improved performance (Schapire, 1999). This approach is closely related to additive models and maximum likelihood estimation (Friedman et al., 2000).

Unlike bagging, which is a parallel ensemble method, boosting is sequential. The weights *w*_*i*_ in Equation 2 depend on the functions previously fitted ${\hat{f}}_{1},\ldots ,{\hat{f}}_{i-1}$. Boosting does not rely on bootstrap sampling; instead, each tree is trained on a modified version of the data.

A widely used boosting algorithm is AdaBoost (Freund and Schapire, 1996), which has been shown to be very effective in classification. For regression, gradient boosting has been proposed (Friedman, 2001, 2002). In this article, we rely on the gradient boosting algorithm of Chen and Guestrin (2016), which uses regression trees as basis functions and optimizes the following regularized objective function:


$$\begin{array}{l} L\left(\phi \right)=\underset{i}{\sum }l\left({ŷ}_{i},y_{i}\right)+\underset{b}{\sum }\text{Ω}\left(f_{b}\right),\text{ Ω}\left(f_{b}\right)=\gamma T+\frac{1}{2}\text{λ}∥w{∥}^{2}, \end{array}$$

where *l* is a differentiable convex loss function measuring the difference between the prediction ŷ_*i*_ and the true response *y*_*i*_, and Ω penalizes the complexity of the model. The regularization term helps smooth the learned weights and prevents overfitting.

At each iteration *m*, a new weak learner *h*_*m*_(*x*) is fitted to the negative gradient of the loss function with respect to the current model:


$$r_{im}=-{\left[\frac{\partial L\left(y_{i},f\left(x_{i}\right)\right)}{\partial f\left(x_{i}\right)}\right]}_{f\left(x\right)=f_{m-1}\left(x\right)}.$$

The model is then updated as:


$$f_{m}\left(x\right)=f_{m-1}\left(x\right)+\nu \cdot h_{m}\left(x\right),$$

where ν ∈ (0, 1) is the learning rate controlling the contribution of each weak learner. This additive optimization process effectively performs a gradient descent in function space, minimizing the empirical risk:


$$\hat{f}=\arg\underset{f\in F}{\min}\overset{n}{\underset{i=1}{\sum }}L\left(y_{i},f\left(x_{i}\right)\right).$$

Thus, boosting reduces both bias and variance by iteratively refining weak learners toward the global minimum of the loss function.

Boosted regression trees combine the advantages of tree-based models with improved predictive accuracy (Friedman and Meulman, 2003).

#### 3.2.4 Model training and hyper-parameter tuning

To ensure reproducibility and fair comparison, all machine learning models were trained using the same training dataset (70%) and evaluated on a hold-out test dataset (30%). Hyperparameters were selected using a grid search with 5-fold cross-validation based on the minimum validation RMSE. For the random forest model, the number of trees (ntree = 300–800), number of variables randomly selected at each split (mtry = 3–8), and maximum tree depth (5–20) were tuned. For bagging, the primary tuning parameter was the number of trees (200–800). For gradient boosting and XGBoost models, the learning rate (0.01–0.1), number of trees (200–600), maximum depth (3–8), and subsampling ratio (0.6–1.0) were optimized using grid search with cross-validation. Categorical predictors were converted into dummy variables using one-hot encoding prior to model fitting. Continuous variables were retained on their original scale unless transformation improved numerical stability. All models were implemented in R using standard machine learning libraries. Final model performance was evaluated using out-of-sample RMSE computed on the test dataset to ensure unbiased predictive comparison.

## 4 Application

### 4.1 Tweedie model

The Tweedie regression model was applied to the insurance dataset to model claim severity conditional on claim occurrence. As described in the data preprocessing stage, the original dataset contained a large proportion of zero claims. Since the objective of this study is to evaluate predictive performance for positive claim amounts, all zero-claim observations were excluded prior to modeling. Consequently, the response variable claim size consists only of strictly positive continuous claim values. Within this conditional severity framework, the Tweedie generalized linear model (GLM) provides a flexible parametric approach for modeling skewed and heteroscedastic positive claim data. Although the Tweedie family is often used in actuarial applications for compound frequency–severity modeling, in this study, it is employed as a benchmark model for positive claim severity to facilitate comparison with machine learning approaches. A sequence of Tweedie GLMs was fitted for a range of power parameters *p* = (1.9, 2,2.5,3,4,5) to examine sensitivity and model stability. For each fitted model, dispersion, null deviance, residual deviance, Akaike Information Criterion (AIC), and root mean square error (RMSE) were computed to assess model fit and predictive performance.

Although RMSE values remained nearly constant across models, indicating stable predictive performance, the deviance and AIC values showed substantial improvement around *p* = 2.0 to *p* = 2.5. Hence, the model with *p* = 2.5 was selected as the optimal Tweedie model for this dataset (Table 3).

**Table 3:** Model comparison of Tweedie GLMs with varying power parameters.

**Power (*p*)**	**Dispersion**	**Null deviance**	**Residual deviance**	**AIC**	**RMSE**
1.9	0.0188876	1,221.3151	1,003.6565	10,484,265	395,282.5
2.0	0.0148749	961.0064	789.8181	7,680,744	395,295.0
2.5	0.0045084	290.1592	238.6310	1,448,798	395,358.0
3.0	0.0013674	87.7479	72.2332	1,520,157	395,421.9
4.0	0.0001261	8.0628	6.6550	1,794,009	395,552.6
5.0	0.0000117	0.7455	0.6176	1,940,867	395,687.6

Table 4 presents the estimated coefficients from the optimal Tweedie regression model with *p* = 2.5, applied to insurance claim data. The positive intercept (${\hat{\beta }}_{0}=2.258$) reflects the baseline log-claim amount when all predictors are at their reference levels.

**Table 4:** Results of the Tweedie regression model (*p* = 2.5) applied to insurance claim data.

**Variable**	**Estimate**	**Standard error**	***t*-value**	***p*-value**
(Intercept)	2.258	0.160	14.115	< 2e-16
Sex (male)	0.0362	0.0016	21.964	< 2e-16
Sex (female)	0.0296	0.0024	12.421	< 2e-16
Insurance type (commercial)	0.0249	0.0067	3.747	0.000179
Insurance type (bus)	–0.0060	0.0052	–1.156	0.247
Insurance type(others)	0.0026	0.0051	0.518	0.605
Insurance type(pick-up)	–0.0428	0.0060	–7.090	1.36e-12
Insurance type(station wagon)	0.0140	0.0023	6.205	5.49e-10
Insurance type (truck)	–0.0281	0.0060	–4.687	2.78e-06
Usage (Goods cartage)	0.0499	0.0048	10.409	< 2e-16
Usage (Other goods)	0.0157	0.0053	2.963	0.00305
Usage (Other service)	–0.0388	0.0028	–14.085	< 2e-16
Usage (others)	–0.0414	0.0040	–10.412	< 2e-16
Usage (private)	–0.0364	0.0080	–4.530	5.91e-06
Make (Isuzu)	0.0300	0.0036	8.230	< 2e-16
Make (Iveco)	0.0134	0.0044	3.075	0.00211
Make (Nissan)	0.0281	0.0037	7.644	2.14e-14
Make (others)	0.0222	0.0031	7.039	1.96e-12
Make (Toyota)	0.0311	0.0031	10.148	< 2e-16
coverage (liability)	–0.0897	0.0021	–43.650	< 2e-16
Production year	0.000037	0.000079	0.468	0.640
Insured value	–6.55e-09	1.13e-09	–5.779	7.56e-09
Premium	1.17e-06	7.50e-08	15.560	< 2e-16

Sex and insurance type were significant predictors of claim amounts. Male policyholders were associated with slightly higher expected claims compared to females, while commercial insurance holders tended to file higher claims than those with other insurance types.

Vehicle characteristics also played a substantial role. Compared to the reference category, station wagons and buses exhibited higher average claim amounts, while pick-ups and trucks were associated with lower claims, indicating possible differences in vehicle risk exposure and usage intensity.

Vehicle usage types such as “Goods Cartage” and “Other Goods” were associated with higher claim costs, suggesting that vehicles used for goods transport are more likely to result in larger or more frequent claims. In contrast, vehicles categorized under “Private” and “Other Services” showed significantly lower claim magnitudes, indicating reduced exposure to high-risk driving environments.

Similarly, vehicle make was a strong determinant of claim size. Vehicles manufactured by Toyota, Isuzu, Nissan, and similar brands exhibited significantly higher expected claim amounts, potentially due to higher replacement and repair costs.

Among the continuous variables, the premium amount was positively associated with claim size, suggesting that higher premiums correspond to larger expected claims. Conversely, insured value showed a small but statistically significant negative relationship, possibly due to conservative claim behavior among holders of high-value vehicles.

Overall, most covariates were highly significant (*p* < 0.001), confirming that the Tweedie GLM effectively captures the variability in insurance claim amounts. The stable RMSE across models and the meaningful directionality of effects further confirm the model's reliability and robustness for mixed discrete–continuous insurance data.

### 4.2 Machine learning methods

#### 4.2.1 Variable importance

Our goal is not only to find the most accurate model of the response, but also to identify which of the predictor variables are most important to make the predictions. For this reason, we perform variable importance. The ensemble methods algorithm estimates the importance of, for instance, *x*_*j*_ predictor variable by looking at how much prediction error increases over the baseline error, when the OOB sample for *x*_*j*_ predictor is permuted while all others are left unchanged. The most commonly used variable importance measure is the permutation importance, introduced by (Breiman 2001), which suggests that the variable importance of predictor *x*_*j*_ is the difference in prediction accuracy after and before permuting *x*_*j*_ averaged over all trees. More precisely, the variable importance of predictor *x*_*j*_ is defined as


$$\begin{array}{l} \text{VI}\left(x_{j}\right)=\frac{1}{B}\overset{B}{\underset{i=1}{\sum }}\text{VI}{\left(x_{j}\right)}_{i} \end{array}$$ (3)

where $\text{VI}{\left(x_{j}\right)}_{i}=\left({\text{RMSE}}_{i}^{x_{j}}-{\text{RMSE}}_{i}^{0}\right)$, and ${\text{RMSE}}_{i}^{x_{j}}$ representing the RMSE value for the *i*^*th*^ model in the ensemble fitted from the dataset with a random permutation applied to the covariate *x*_*j*_, and ${\text{RMSE}}_{i}^{0}$ is the RMSE value for this model fitted to the original dataset. Note that VI(_*x*_*j*_)*i*_ = 0, if variable *x*_*j*_ is not in the *i*^*th*^ model. The raw variable importance score for each variable is then computed as the mean importance over all trees. In fact, VI(*x*_*j*_) can be computed depending on any other performance measure, such as the coefficient of determination, *R*^2^.

Let *x*_1_, …, *x*_*p*_ be the features of interest and let RMSE_0_ be the baseline performance metric for the trained model. The permutation-based variable importance scores can be computed as shown in Algorithm 1.

Algorithm 1 **Permutation-based variable importance computation.**

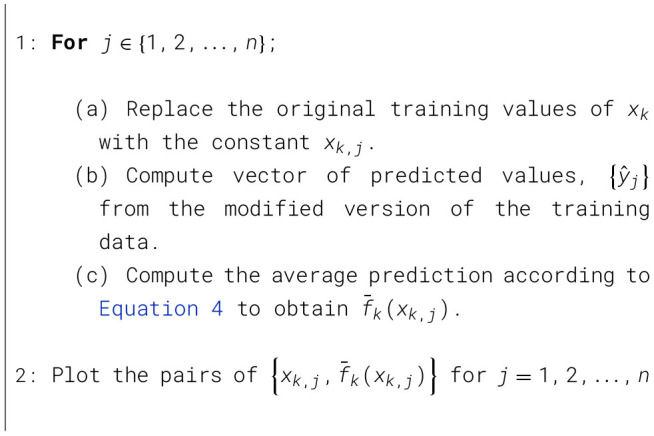

**Figure 4:**
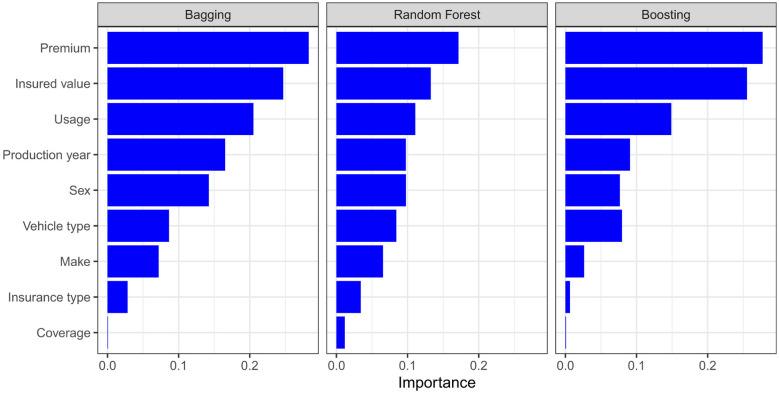
A graphical representation of average variable importance across all the trees from Bagging, boosting, and regression RF. The larger the number, the bigger the effect.

Figure 4 displays the importance predictor while growing the trees. Accordingly, in all three models, premium is the most crucial predictor, followed by the insured value. The second most influential (slightly equally in bagging and random forest) predictors are usage and sex, followed by production year, in line with the earlier boxplots exploratory analysis.

**Figure 5:**
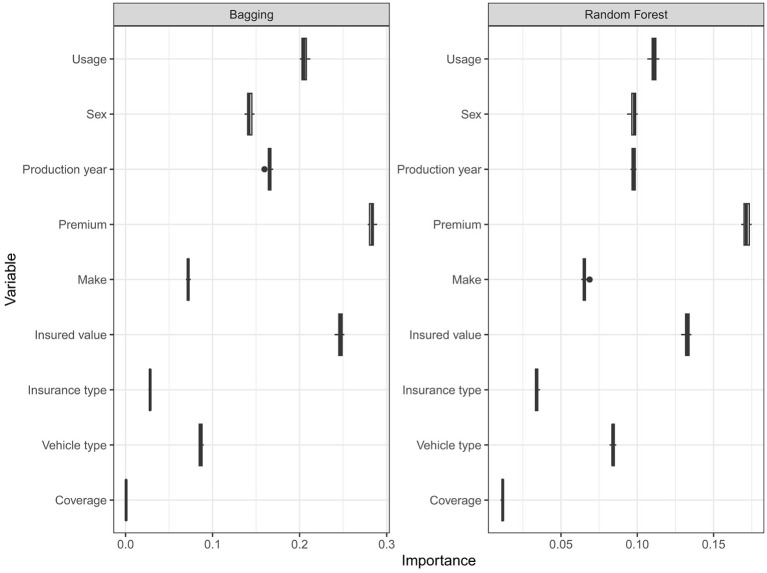
Boxplots of variable importance from Bagging and regression random forest from 20 times repeated permutation.

Figure 4 is obtained by repeating the permutation of each variable 20 times and the results are averaged together. This helps to provide more stable VI scores, and also the opportunity to measure their variability as seen in Figure 5, since the permutation approach introduces randomness into the procedure.

#### 4.2.2 Partial dependence plots

Though determining predictor importance is a crucial task in any supervised learning problem, ranking variables is only part of the story, and once the important predictors are identified, it is often necessary to assess the relationship between the predictors and the response variable. The task is often accomplished by constructing partial dependence plots (PDP) (Friedman, 2001), which helps to visualize the relationship between a predictor and the response variable while accounting for the average effect of the other predictors in the model.

Let ŷ be a prediction function from an arbitrary model using a dataset, *D* = {(*x*_*i, j*_, *y*_*i*_)} for *i* = 1, …, *n* and *j* = 1, …, *p*. The model generates predictions of the form:


$$\begin{array}{l} {ŷ}_{i}=f\left(x_{i,1},x_{i,2},\ldots ,x_{i,p}\right), \end{array}$$

for some function *f*(...).

Let *x*_*k*_ be a single predictor of interest with unique values (*x*_1, *k*_, *x*_2, *k*_, …, *x*_*n, k*_). Then the partial dependence plots are obtained by computing the following average and plotting it over a useful range of *x* values:


$$\begin{array}{l} {\bar{f}}_{k}\left(x\right)=\frac{1}{n}\overset{n}{\underset{j=1}{\sum }}\hat{f}\left(x_{1,j},\ldots ,x_{k-1,j},x,x_{k+1,j},\ldots ,x_{p,j}\right) \end{array}$$ (4)

The function ${\bar{f}}_{k}\left(x\right)$ indicates how the value of the variable *x*_*k*_ influences the model predictions {*y*_*j*_} after we have averaged out the influence of all other variables. The partial dependence of the response on *x*_*k*_ can be constructed as Algorithm 2:

Algorithm 2 **Partial dependence construction of the response on a single predictor *x*_*k*_.**

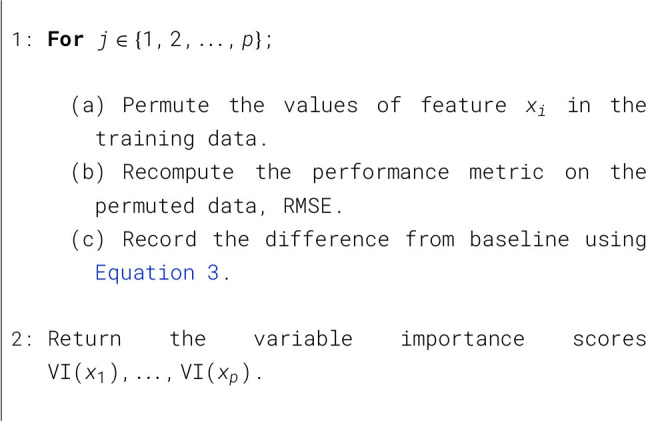

Since Algorithm 2 can be quite computationally intensive as it involves *n* passes over the training records, a reduced number of points is used by equally spacing the values in the range of the variable of interest.

**Figure 6:**
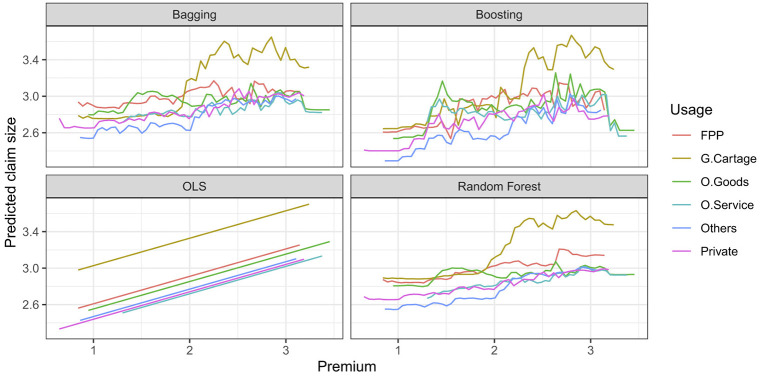
Two-way partial dependence plots; the marginal effect of premium on the claim size for different groups of usage predictor after integrating out the other variables.

Figure 6 shows a separate partial dependency function for each group of usage predictors. Because one-way partial dependency plots display one predictor at a time, they are valid only if the predictor of interest does not interact strongly with other predictors. However, interactions are common in actual practice; in these cases, we can use higher order (such as two- and three-way) partial dependence plots to check for interactions among predictors. For example, Figure 6 shows an interaction between premium and usage predictors.

The two-way plot shows that vehicles used for general cartage with a high premium (more than 2 in ensemble methods) have much higher expected claim sizes than vehicles with other usages. This interaction would not have been apparent in the one-way plot.

### 4.3 Methods comparison

In this application, the predictor variable is represented by a collection of quantitative and qualitative attributes of the vehicle, and the response is the actual claim size. Given a collection of *N* observations {(*x*_*i*_, *y*_*i*_);*i* = 1, …, *N*} of known (*x, y*) values, the goal is to use this data to obtain an estimate of the function that maps the predictor vector *x* to the values of the response variable *y*. This function can then be used to make predictions for observations in which only the *x* values are observed. Formally, we wish to learn a prediction function $x\mapsto \hat{f}\left(x\right)$ that minimizes the expectation of some loss function $L\left(\hat{f}\left(x\right),y\right)$ over the joint distribution of all (*x, y*) values, that is,


$$\begin{array}{l} \hat{f}\left(x\right)=argmin_{f\left(x\right)}E\left[L\left(f\left(x\right),Y\right)|X=x\right]. \end{array}$$

In finite samples, we evaluate the performance of $\hat{f}$ with the mean square error (MSE), that is,


$$\begin{array}{l} \text{MSE}=\frac{1}{n^{\prime }}\overset{n^{\prime }}{\underset{i=1}{\sum }}{\left(\hat{f}\left(x_{i}\right)-y_{i}\right)}^{2} \end{array}$$

where $\hat{f}\left(x\right)$ is a fitted regression function in the test dataset ${\left\{x_{i}\right\}}_{i=1}^{n^{\prime }}$ and *y* is the observed response variable.

For statistical modeling purposes, we first partitioned the data into train (70%) and test (30%) datasets. The train set was used for exploratory data analysis, model training, and selection, and the test set was used to assess the predictive accuracy of the selected method. The training data goes from 2011 to 2015 and from 2017 to 2018, while the test data is observations from 2016.

Table 5 presents the predictive accuracy of the five models based on their root mean square error (RMSE) values. The *Random Forest* model achieved the lowest RMSE (656.1), indicating slightly superior predictive accuracy compared to the other models. In contrast, the *Tweedie* regression recorded the highest RMSE (663.9), suggesting relatively weaker predictive power. Overall, the results imply that the ensemble learning methods offered only marginal improvements over the Tweedie model, reflecting limited non-linearity and interaction effects in the vehicle insurance claim data.

**Table 5:** Predictive performance comparison based on RMSE values.

Model	Tweedie (*p* = 2.5)	Random forest	Bagging	Boosting	OLS
RMSE	663.9	656.1	657.5	658.6	662.9

**Figure 7:**
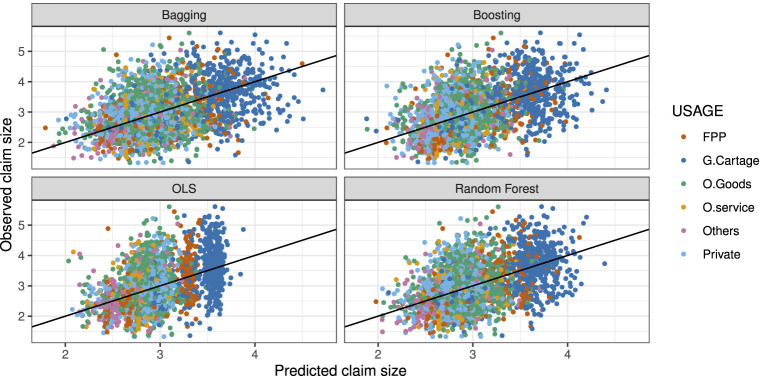
Observed against predicted claim size on log scales.

Regarding the performance of the methods, it can be clearly seen in Figure 7 that the ordinary least squares (OLS) method is predicting all the claims less than USD 10^4^ even though some observed claims are even larger than USD 10^5^. However, in the case of ensemble methods, they could predict claim size beyond 10^4^. Both OLS and ensemble methods underestimated high claim size, but the underestimation is higher in OLS.

## 5 Discussion and conclusions

This study provided a structured comparative evaluation of classical statistical models and modern ensemble learning techniques for predicting vehicle insurance claim severity. Rather than introducing a novel methodological framework, the primary objective was to assess the predictive performance, stability, and interpretability of established statistical and machine learning approaches when applied to real-world insurance data within a conditional severity setting.

The analysis was conducted on strictly positive claim size, thereby focusing on conditional mean severity rather than the full frequency–severity process. This distinction is important when interpreting the role of parametric distributions such as the Tweedie model, which was employed here as a flexible benchmark for positive and heteroscedastic claim data rather than as a compound loss model.

Empirical results indicate that ensemble learning methods–bagging, random forest, and gradient boosting–achieved slightly lower RMSE values than ordinary least squares (OLS) and the Tweedie generalized linear model. However, the magnitude of improvement was modest, and performance differences among the ensemble techniques themselves were relatively small. These findings suggest that while machine learning methods can capture non-linearities and interactions, the underlying dataset structure may not exhibit sufficiently strong complexity to yield large predictive gains over well-specified parametric models.

The Tweedie regression model, widely used in actuarial science due to its capacity to model skewed and heteroscedastic claim distributions, demonstrated competitive performance and remains a robust and interpretable benchmark for conditional severity modeling. This result aligns with broader actuarial evidence that flexible parametric models often remain effective when model assumptions are not severely violated.

Variable importance analysis showed that premium amount and insured value were consistently the most influential predictors of claim severity across ensemble models. Additional predictors–including vehicle usage, production year, and vehicle type–also contributed meaningfully to predictive performance. Partial dependence analysis, as formalized by Friedman (2001), provided further insight into the marginal effects of these predictors while averaging over other covariates. The findings underscore the central role of financial exposure measures and vehicle characteristics in determining claim outcomes.

From a methodological perspective, the results complement recent actuarial research on tree-based methods in insurance. While studies such as Janoušek and PeŽta (2025) emphasize predictive stability and robustness in individual-claims reserving, and (Maciak et al. 2022) highlight structured and functional modeling of claim development processes, the present study demonstrates that similar ensemble techniques can be effectively applied in a cross-sectional conditional severity context. However, unlike reserving frameworks that explicitly model temporal claim development and outstanding liabilities, the current analysis does not incorporate longitudinal structure. Therefore, insights regarding stability and interpretability carry over, whereas structured development modeling does not directly apply in this setting.

Despite the reasonable predictive performance achieved, the modeling approaches considered here focus on conditional mean estimation and do not explicitly address tail risk. In insurance portfolios, a small number of extreme claims can disproportionately affect aggregate losses. Accurate modeling of the upper tail remains essential for capital adequacy and solvency assessment. Future research should therefore explore advanced frameworks such as quantile regression for conditional tail modeling (Koenker and F.Hallock, 2001) and hybrid approaches that combine machine learning methods with Extreme Value Theory. Recent contributions have demonstrated the usefulness of integrating generalized random forests with the Generalized Pareto Distribution for tail-risk modeling in insurance and finance (Gnecco et al., 2024; Pasche and Engelke, 2024; Velthoen et al., 2023; Youngman, 2019; Athey et al., 2019).

In conclusion, this study contributes empirical evidence on the comparative behavior of statistical and machine learning approaches for motor insurance claim severity modeling using data from an emerging market context. While ensemble methods achieved marginal improvements in predictive accuracy, traditional parametric models remained competitive and interpretable. The results suggest that the choice between statistical and machine learning approaches should depend on the balance between interpretability, stability, and incremental predictive gain, rather than on an assumption of universal superiority of one class of methods over another.

## Author's note

A preprint of this article has previously been published (Terefe, 2023).

## Data Availability

The datasets presented in this study can be found in online repositories. The names of the repository/repositories and accession number(s) can be found below: https://data.mendeley.com/datasets/34nfrk36dt/1.
